# Miro1 – the missing link to peroxisome motility

**DOI:** 10.1080/19420889.2018.1526573

**Published:** 2018-10-12

**Authors:** Inês G. Castro, Michael Schrader

**Affiliations:** a Department of Molecular Genetics, Weizmann Institute of Science, Rehovot, Israel; b Biosciences, University of Exeter, Exeter, UK

**Keywords:** Miro1, peroxisome, organelle motility, microtubule, actin

## Abstract

Peroxisomes are ubiquitous, highly dynamic, multifunctional compartments in eukaryotic cells, which perform key roles in cellular lipid metabolism and redox balance. Like other membrane-bound organelles, peroxisomes must move in the cellular landscape to perform localized functions, interact with other organelles and to properly distribute during cell division. However, our current knowledge of peroxisome motility in mammalian cells is still very limited. Recently, three independent studies have identified Miro1 as a regulator of peroxisome motility in mammalian cells. In these studies, the authors show that Miro1 is targeted to peroxisomes in several cell lines, in a process that relies on its interaction with the peroxisomal chaperone Pex19. Interestingly, however, different conclusions are drawn about which Miro1 isoforms are targeted to peroxisomes, how it interacts with Pex19 and most importantly, the type of motility Miro1 is regulating.

Peroxisomes are highly dynamic and multifunctional organelles which perform several cellular roles such as fatty-acid α- and β-oxidation, ether phospholipid biosynthesis and reactive oxygen species (ROS) metabolism [1]. Due to their many functions, peroxisomes play an essential role in human health and development. Consequently, defects in genes that encode peroxisomal proteins lead to severe neurological and developmental diseases, such as Zellweger spectrum disorders (reviewed in [2]). Furthermore, due to their central roles in cellular energy and lipid metabolism, peroxisome dysfunction contributes to aging, cancer and several neurodegenerative disorders, such as Alzheimer’s and Parkinson’s disease [3–5].

Peroxisomes respond to fluctuations in cellular nutritional and environmental states by changing their number, morphology and function [6,7]. To enable these adaptations, peroxisomes move along cytoskeletal tracks, a process which likely facilitates their metabolic interaction with other organelles and assists in their membrane expansion and division pathways [8].

In mammalian cells, peroxisomes move along microtubules using kinesin and dynein motors (reviewed in [9,10]). Strikingly, little is known about the recruiting factors for these motors and how peroxisome motility is regulated. Recently, we and others have shown that Miro1, an atypical Ras GTPase, is targeted to peroxisomes in mammalian cells, where it regulates peroxisome motility (Figure 1) [11–14].

Miro proteins (Miro1 and Miro2 in mammals) are membrane proteins that were initially identified and characterized on the outer mitochondrial membrane, and which are highly conserved across eukaryotes [15,16]. These atypical Ras GTPases contain two GTPase domains separated by two calcium-binding EF-hand motifs, and a C-terminal transmembrane domain (TMD) and short tail that anchors them to the cytosolic side of the organelle’s membrane. Miro1 in particular has been extensively studied in mammalian cells, where it forms a motility complex with TRAK1 and TRAK2, and both kinesin and dynein motors (Figure 1) [17–21]. Additionally, due to its calcium binding motifs, Miro1 has been proposed to inhibit mitochondrial motility in active synapses, where glutamate signalling induces high intracellular calcium concentrations [18,21], and to regulate mitochondrial Ca^2+^ storage in response to cytosolic fluctuations [22].

**Figure 1.**
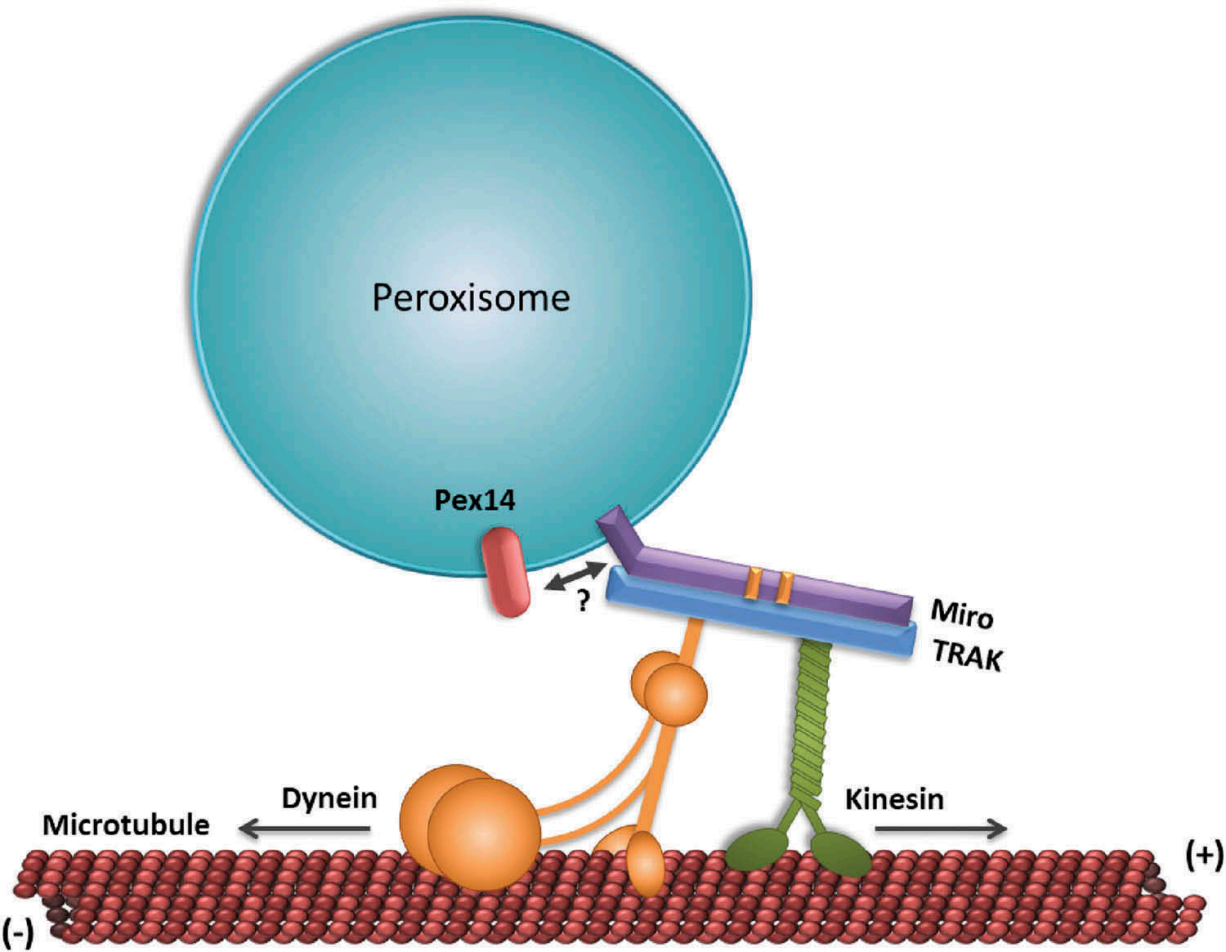
A model showing components of the peroxisomal motility complex in mammals. The tail-anchored membrane protein Miro is targeted to peroxisomes via Pex19 (see text). Miro forms a complex with TRAK, and both microtubule minus (dynein) and plus-end (kinesin) directed motor proteins. The peroxisomal membrane protein Pex14 may function as a microtubule docking factor (Castro *et al*., 2018). Other Miro interacting proteins at peroxisomes await identification.

## Targeting and Pex19 binding

Miro proteins have a single TMD at their C-terminus followed by a short amino acid tail that protrudes into the organelle lumen (Figure 1). As a result of this tail-anchored (TA) topology, these proteins are post-translationally directed to their target membranes. Interestingly, numerous TA proteins are dually targeted to both peroxisomes and mitochondria [23]. This targeting appears to be largely dependent on the biochemical properties of their C-terminal amino acid sequence (principally the hydrophobicity of the TMD and the net charge in the tail region) and, as such, it can be characterized and used to predict the targeting of TA proteins [12]. Interestingly, Miro1 has an identical TMD hydrophobicity and tail charge to that of Mff, another dually targeted protein involved in mitochondrial and peroxisomal division [24].

Several TA proteins have been shown to interact with Pex19, a cytosolic chaperone involved in the targeting of membrane proteins to peroxisomes. As shown in all three studies, Miro1 is no exception to this rule [11,13,14]. However, the conclusions on how this interaction is mediated and which isoforms of Miro1 are targeted to peroxisomes differ between studies. Okumoto and colleagues characterized four Miro1 isoforms (named variants 1–4) and show that in HeLa cells only variants 2 and 4 are targeted to peroxisomes [13]. According to the authors, this targeting strongly relies on the presence of a short amino acid sequence – insert 1 – that is present in both variants and contains a predicted Pex19 binding site. Specific mutations to this binding site strongly affected the targeting of variants 2 and 4 to peroxisomes. Additionally, and in line with our current knowledge of TA protein targeting signals, the authors also show an influence of the charge of the C-terminal tail on Pex19 binding and targeting to peroxisomes.

In contrast to these findings, we and Covill-Cooke and colleagues observed the targeting of Miro1 variant 1 to peroxisomes and mitochondria in COS-7 and mouse embryonic fibroblasts (MEFs) [11,14]. This variant, which lacks insert 1, was also shown to interact with Pex19 in a TMD and tail dependent manner. Furthermore, Covill-Cooke and colleagues showed that the first GTPase domain of Miro1 might also play a role in its ability to interact with Pex19, as expression of a Miro1 truncated protein lacking this domain results in increased peroxisomal localization [14]. These discrepancies might result from the use of different cell lines or the use of different experimental conditions, e.g. Miro1 expression levels. It should also be noted that Okumoto and colleagues used a truncated form of Miro1 (Flag-GFP-insert-TMD-tail) for most of their experiments with Pex19. As shown by the authors, the truncated versions of variant 2 and 4 have a significantly decreased targeting to peroxisomes when compared to the full length protein, substantiating the influence of other targeting information in the transport to peroxisomes. All together, these results suggest that additional factors are at play regarding Miro1 targeting to peroxisomes and its interaction with Pex19.

## Miro1 and peroxisome motility

Having established that Miro1 is targeted to peroxisomes in mammalian cells, the question then becomes: what is its function on this organelle? Overexpression of Miro1 (wild-type and constructs mutated in the first GTPase domain) in COS-7 and HeLa cells induced a redistribution of peroxisomes to the cell periphery, an effect that was inhibited by treating the cells with nocodazole, a microtubule depolymerizing drug [11,13]. These results suggest a potential role for Miro1 in peroxisome motility, similar to its role in mitochondrial motility (Figure 1). In agreement with this, similar peroxisome accumulations have been previously observed using an inducible cargo assay where kinesin motors are artificially tagged to peroxisomes in a regulated and inducible manner [25]. Furthermore, overexpression of wild type Miro1 or a constitutively active mutant for the 1^st^ GTPase domain of Miro1, induced a significant increase in peroxisome fast-directed movement [11,13], while silencing of Miro1 significantly decreased peroxisome motility [13]. These results suggest that, analogous to Miro1’s role at mitochondria, peroxisomal Miro1 regulates microtubule-dependent motility of this organelle (Figure 1).

However, analysis of MEFs from Miro1 knockout mice [11,26] or double knockouts for Miro1 and Miro2 [14] showed no changes in peroxisome distribution and fast directed motility in cells, despite clear effects on the mitochondrial network. Instead, Covill-Cooke and colleagues observed a decrease in peroxisome oscillatory movement. This type of short-range motility accounts for 85–95% of peroxisome movements in the cell, and although poorly characterized, might rely on diffusive behaviour and interactions with the actin cytoskeleton [27,28]. Although conflicting with the expression and silencing results, it is possible that in the absence of Miro1, other motor complexes enable fast and directed motility of peroxisomes, but also that Miro1 plays additional roles in peroxisome motility.

## Conclusions

Taken together, these results show a new role for Miro1 in mammalian cells and further strengthen the connection between peroxisomal and mitochondrial regulatory pathways. In light of these results and taking into account the discrepancies between studies, it will be very interesting to further analyse the role of Miro1 (and Miro2) on peroxisomes, and to better understand the functions of Miro and its binding partners. Ultimately, an improved understanding and ability to manipulate peroxisome motility should enable us to study its importance in the cellular environment and its involvement in regulating cooperative peroxisome functions.
